# Editorial: Use of bioinformatics in pharmacogenetics to optimize drug efficacy

**DOI:** 10.3389/fphar.2026.1930155

**Published:** 2026-07-30

**Authors:** Abdeslam Jaafari, Mounir Tilaoui

**Affiliations:** 1 Sultan Moulay Slimane University, Béni Mellal, Morocco; 2 Laboratory of Biotechnology, Environment, Agrifood, and Health, Department of Biology, Faculty of Sciences Dhar El Mahraz, Sidi Mohamed Ben Abdellah University, Fez, Morocco

**Keywords:** bioinformatics, data analysis, drug efficacy, pharmacogenetics, precision medecine

Pharmacogenetics evaluates the relationship between drug efficacy and pharmacogenes. The latter are a set of genes that control variation in drug pharmacokinetic and pharmacodynamic characteristics (Borbón et al., 2025). Any alteration in their expression could lead to treatment failure and/or an increase in adverse drug effects (Asghar et al., 2025). The large number of pharmacogenes and therapeutic agents necessitates the use of high-performance bioinformatics approaches to analyze the large volumes of data generated by next-generation sequencing technologies. Indeed, Bioinformatic tools play a key role in the extraction, annotation, aggregation, and integration of data. They also can help in clinical interpretation and implementation (Pereira et al., 2020; Dezso and Ceccarelli, 2020). The goal is to optimize and improve drug efficacy within the framework of personalized medicine, which means “giving the right drug to the right patient at the right dose” (Navarro et al., 2024).

In this editorial, we shed light on this Research Topic by providing an overview of some recent discoveries in the field of using Bioinformatic tools in pharmacogenetics to optimize drug efficacy. After the analysis of eleven articles published in our Research Topic, it was reported that Bioinformatic tools could be used in different ways with the aim to optimize drug efficacy (by increasing activity and decreasing adverse effects) through Pharmacogenetic data analysis.

In a study aiming to explore the purine metabolism-related gene signatures in the context of immunotherapeutic strategies for nonspecific orbital inflammation (NSOI), Wu et al. reported that seven pharmacogenes (ENTPD1, POLR2K, NPR2, PDE6D, PDE6H, PDE4B, and ALLC) were intimately connected to NSOI through their involvement in processes such as peroxisome targeting, sequence binding, seminiferous tubule development, and ciliary transition zone organization. The researchers used advanced methodologies, including Gene Set Enrichment Analysis (GSEA) and Gene Set Variation Analysis (GSVA) to explore the biological functions and pathways associated with these pharmacogenes. Furthermore, Lasso regression and Support Vector Machine-Recursive Feature Elimination (SVM-RFE) enabled the identification of key hub genes and the evaluation of their diagnostic interest for NSOI (Wu et al.). Other Bioinformatic tools were also used by Dashti et al. to determine HLA-B allele frequencies and implications for pharmacogenetics in the Kuwaiti population. 561 Kuwaiti individuals were sequenced on the Illumina HiSeq platform and the HLA typing was conducted using the HLA-HD tool with a reference panel from the IPD-IMGT/HLA database. The major HLA-B pharmacogenetic markers were obtained from the HLA Adverse Drug Reaction Database. This study provides important information to use in pharmacogenetic research and potential personalized medicine (Dashti et al.). A pharmacogenetic study aiming to find out the association between genetic variation and drug efficacy was performed by Durán-Sotuela et al. using appropriate statistical analyses and internal validation, including logistic regression models to assess the impact of these genetic variants in the efficacy of Tocilizumab in terms of mortality. The results reported that, in patients treated with Tocilizumab, the presence of some genotypes like GG and TT at IL10Rβ (rs2834167) and CC at IL1RN (rs2234679) is significantly associated with a reduced or increased risk of mortality, respectively. In this study, the accuracy of the predictive models was using a bootstrap method (B = 500 replicates) (Durán-Sotuela et al.).

Bioinformatic tools were also used to analyze a transcriptome in order to explore the relationship between TNIK regulation and the effect of Risperidone. Transcriptome analysis was performed on U251 cells subjected to Risperidone, TNIK siRNA, using GO and KEGG. On the other hand, STRING and Cytoscape was performed to construct Protein-protein interaction (PPI) network for the cross-talk gene (Yuan et al.). GO and KEGG are standard Bioinformatic methods used to interpret “omics” data such as RNA-seq or microarray results by evaluating expression of genes involved in different functions and pathways (Huang et al., 2025).

In another study, a tripartite network approach that combines drug-target and target-phenotype data was conducted to analyze the phenotypic effects of drugs through shared targets in genetic disease networks like cancer (Díaz-Santiago et al.). Researchers also used Bioinformatic tools to predict side effects and novel targets of drugs and drug combinations (Dezso and Ceccarelli, 2020). Indeed, Lachmann et al. used the L1000 assay to profile transcriptional responses to 11 single ART drugs and 6 ART combination regimens to analyze differentially expressed genes against host-HIV PPIs and genes implicated in ART-associated side effects (Lachmann et al.). These tools are also used for genome-wide functional annotation and interpretation of variants and other data generated by new technologies like NGS (Pilalis et al.). Regarding drug metabolism enzymes which are very important in Pharmacogenetics, a study using the National Institute of Health All of Us Research Program Data Browser, differential distribution of CYP2D6 alleles with ClinVar “drug response” was performed to improve population-specific drug response knowledge (Hendricks-Sturrup et al.).

In an interesting study, a personalized genome interpretation workflow was developed based on open-source code for facilitating the practice of precision medicine. Two validated pharmacogene panels were used (the 12-pharmacogene PREPARE study panel and the 87-pharmacogene PyPGx panel). The research team analyzed and reported clinically actionable, rare and novel variants. This workflow holds promise to facilitate implementation of pharmacogenetics in the clinic using NGS data (Patrinos et al.). Furthermore, to explore the causal effects of statin medication on gut microbiota abundance, Mendelian randomization study using GWAS (Genome-Wide Association Studies) data analysis was used. In this study, Statin medication could be negatively or positively correlated with different species of gut bacteria abundance by calculation of BetaIVW (Inverse-Variance Weighted Estimate) and PIVW (Penalized Inverse-Variance Weighted) (Zhou et al.).

In summary, the studies included in this Research Topic illustrate the central role of bioinformatics in advancing pharmacogenetics and pharmacogenomics. The application of genomic databases, computational analyses, and high-throughput technologies has improved the identification of pharmacogenetic biomarkers, the interpretation of genetic variation, and the prediction of drug response and toxicity. Collectively, these findings highlight the growing contribution of bioinformatics to precision medicine and personalized therapy. Continued advances in next-generation sequencing, large-scale genomic resources, and artificial intelligence are expected to further facilitate the clinical implementation of pharmacogenomics, ultimately improving the safety and efficacy of drug treatment.
